# The clinical significance and optimization strategies of HBeAg seroclearance in chronic hepatitis B treatment

**DOI:** 10.3389/fcimb.2026.1785154

**Published:** 2026-06-29

**Authors:** Yaqin Zhang, Yuanjiao Gao, Xinxin Li, Ziyu Zhang, Shiyu Wang, Zixuan Gao, Yao Xie, Minghui Li

**Affiliations:** 1 Department of Hepatology Division 2, Beijing Ditan Hospital, Capital Medical University, Beijing, China; 2 HBV Infection, Clinical Cure and Immunology Joint Laboratory for Clinical Medicine, Capital Medical University, Beijing, China; 3 Department of Hepatology Division 2, Peking University Ditan Teaching Hospital, Beijing, China

**Keywords:** chronic hepatitis B, functional cure, HBeAg, immune, treatment

## Abstract

HBeAg seroclearance represents a critical milestone in the treatment of HBeAg-positive chronic hepatitis B (CHB), which is closely associated with disease remission, functional cure, and reduced hepatocellular carcinoma risk. As HBeAg is a secreted viral protein associated with active HBV replication, infectivity, and host immune tolerance, its disappearance usually indicates a transition toward improved immune control rather than simple loss of a laboratory marker. This review comprehensively explores the immunological mechanisms underlying HBeAg seroclearance, thoroughly examines its impact on the natural course of CHB and systematically analyzes the regulatory roles of antiviral drug efficacy, host immune status, and viral genotypes in HBeAg clearance. Integrating updated guidelines and evidence-based research, we propose optimized strategies for managing HBeAg-negative CHB, including goal setting, treatment cessation criteria, and long-term monitoring protocols. Our findings emphasize that HBeAg clearance not only signifies virological suppression but also reflects the dynamic equilibrium of host immune responses, thereby providing both theoretical foundation and practical support for achieving functional cure and reducing HCC risk in CHB patients.

## 1 Introduction

Hepatitis B virus (HBV) infection remains a major global health threat and one of the leading causes of chronic liver inflammation, cirrhosis, and hepatocellular carcinoma (HCC). Chronic hepatitis B (CHB) results from persistent HBV infection and is characterized by dynamic interactions among viral replication, host immune responses, and liver injury. Epidemiological data reveal the ongoing public health impact of HBV, with 296 million chronic infections worldwide in 2019 (ORGANIZATION W H, 2023).

HBV expresses several clinically important antigens, including hepatitis B surface antigen (HBsAg), hepatitis B core antigen (HBcAg), and hepatitis B e antigen (HBeAg) (Coffin et al., 2019). HBsAg reflects the presence of HBV envelope proteins and is the key marker used to define persistent HBV infection, whereas HBeAg is a soluble, secreted protein translated from the precore/core region. Although HBeAg is not required for viral particle formation, it is closely associated with active viral replication, high infectivity, immune tolerance, and treatment response. HBeAg serves as a critical biomarker for HBV replication and immune regulation, and its seroconversion represents a key event in the natural history of CHB and treatment response evaluation.

In clinical terms, seroclearance refers to the loss of detectable circulating viral antigen, whereas seroconversion is defined as antigen loss accompanied by the emergence of the corresponding antibody. HBeAg seroclearance denotes loss of serum HBeAg, and HBeAg seroconversion is defined as HBeAg loss accompanied by the emergence of anti-HBe. HBsAg seroclearance (with or without anti-HBs seroconversion) represents a deeper state of viral suppression and is widely recognized as the closest clinical endpoint to functional cure in CHB. Despite continuous advancements in antiviral therapies that have substantially reduced HBV DNA levels and brought hope to patients, HBeAg seroclearance rates demonstrate significant variability among patient populations. Even patients achieving HBeAg seroclearance remain vulnerable to disease reactivation and HCC risk, presenting ongoing challenges in CHB management.

Conventional medical perspectives attribute multifaceted clinical significance to HBeAg seroclearance. It not only indicates reduced viral replication and improved host immune control but also suggests enhanced potential for functional cure and significantly lowered HCC risk (Liaw, 2009). However, emerging research reveals more complex realities. Some HBeAg-negative patients develop occult hepatitis progression due to viral immune evasion or mutations, demonstrating that HBeAg negativity does not equate to complete disease control (Bonino et al., 2022). Therefore, HBeAg seroclearance should be interpreted as an important intermediate endpoint: it marks a favorable shift in host-virus balance, but it does not prove elimination of covalently closed circular DNA (cccDNA), integrated HBV DNA, or HBeAg-negative variants driven by precore or basal core promoter mutations. This distinction explains why HBeAg seroclearance is clinically valuable while long-term monitoring remains necessary. In-depth immunological studies reveal that HBeAg exerts multiple functions including immune tolerance induction, T-cell function suppression, and apoptosis regulation–mechanisms crucial for HBV persistence (Lan et al., 2016; Yang et al., 2019; Tsai and Ou, 2021). Therefore, comprehensive understanding HBeAg’s immunological roles and the clinical implications of its seroconversion has become essential for overcoming CHB treatment barriers and optimizing therapeutic strategies.

In recent years, with in-depth research into the mechanisms of HBeAg seroclearance, multiple therapeutic strategies have been proposed to enhance HBeAg clearance rates and functional cure rates. Two common treatment approaches–long-term nucleos(t)ide analogues (NAs) therapy and finite-course pegylated interferon alpha (Peg-IFNα) regimens–demonstrate distinct capacities for inducing HBeAg seroconversion and divergent post-treatment outcomes (Liu et al., 2018; Liem et al., 2019; Lee SK. et al., 2021; Kumar et al., 2024). Confronted with these discrepancies, clinicians urgently require an evidence-based personalized decision-making framework to develop more precise and effective treatment plans for individual patients. Concurrently, therapeutic concepts and cessation criteria for HBeAg-negative patients continue to evolve, aiming to maximize therapeutic efficacy while minimizing relapse risks.

**Figure 1:**
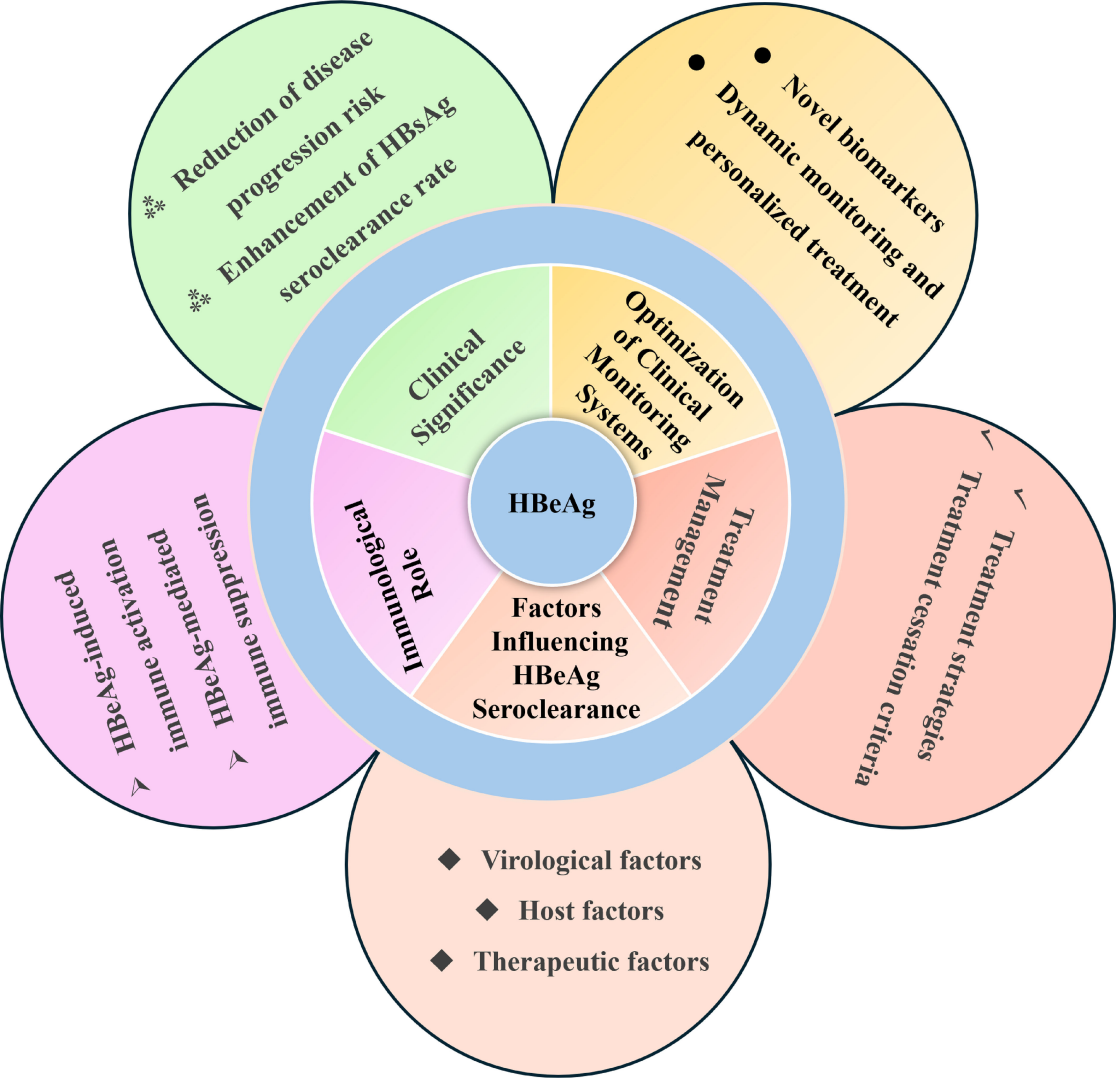
Integrated framework of clinical significance and optimization strategies for HBeAg seroclearance in chronic hepatitis B management.

This article will commence with an exploration of HBeAg’s immunological functions, systematically analyze diverse factors influencing its clearance, investigate optimized treatment pathways, and update post-treatment management paradigms. We aim to provide actionable recommendations for achieving functional cure of CHB and advancing HCC prevention strategies (**Figure 1**).

## 2 The clinical significance of HBeAg seroclearance

### 2.1 Reducing the risk of disease progression

HBeAg seroclearance represents a critical turning point in disease progression for CHB patients, with its achievement closely associated with amelioration of disease severity. Studies demonstrate that after HBeAg seroclearance, patients experience significantly reduced risks of developing liver cirrhosis and HCC.

Clinically, the natural course of chronic hepatitis B (CHB) is divided into distinct dynamic phases based on HBeAg/anti-HBe serostatus, HBV DNA, alanine aminotransferase (ALT) and liver histopathological findings. The HBeAg-positive chronic HBV infection phase presents with positive HBeAg, high viral load, normal or slightly raised ALT, and mild liver necroinflammation. In the HBeAg-positive chronic hepatitis phase, patients have persistent HBeAg positivity, robust viral replication, elevated ALT, as well as histological inflammation and fibrosis. Following HBeAg seroclearance and anti-HBe seroconversion, many patients transition to an inactive state, showing undetectable or low HBV DNA, normal ALT and quiescent liver histology. In contrast, some patients progress to HBeAg-negative chronic hepatitis or the indeterminate phase. Such cases are often associated with precore or basal core promoter mutations, manifesting as fluctuating viral loads, abnormal ALT and ongoing hepatic inflammation. In clinical practice, HBeAg status cannot serve as a standalone indicator and must be evaluated alongside HBV DNA, ALT and liver fibrosis.

According to the 2018 CHB guidelines issued by the American Association for the Study of Liver Diseases (Terrault et al., 2018), the inactive phase is defined as serum HBsAg positivity, HBeAg negativity, anti-HBe positivity or negativity, HBV DNA below 2000 IU/mL, normal ALT levels, and no significant inflammatory necrosis or fibrosis in liver histology; while the disease indeterminate phase refers to not conforming to the traditional phases of hepatitis B virus (immune tolerance phase, immune active phase, inactive phase). A retrospective cohort study of 3,366 untreated adult non-cirrhotic CHB patients found after a mean follow-up of 12.5 years that the 10-year cumulative incidence of HCC in patients in the disease indeterminate phase was as high as 4.6%, compared with only 0.5% in those in the inactive phase, with a significant difference between the two (Huang et al., 2022). This fully indicates that the disease indeterminate phase is a high-risk stage for HCC development. Further research also deeply explored the relationship between different characteristics of patients in the indeterminate phase and the risk of HCC development. Analysis of indeterminate-phase HBeAg-negative patients indicated a higher risk of HCC in the normal ALT/high HBV DNA subgroup compared to the abnormal ALT/low HBV DNA group (Hui et al., 2025). This further reveals the complexity and risk of the disease indeterminate phase. Even within the same disease stage, different combinations of liver function indicators and viral loads can lead to differences in the risk of HCC occurrence.

Of particular importance, relevant studies have indicated that antiviral treatment can decrease the risk of HCC by 70% in CHB patients in the indeterminate phase. Compared with untreated patients, those who received treatment had significantly lower cumulative HCC incidence rates at 10 and 15 years (Huang et al., 2023). Additionally, the latest guidelines for the prevention and treatment of CHB in China have simplified the staging of CHB with clearer classification criteria (You et al., 2023), which has reduced the proportion of patients in the indeterminate phase and is of great significance for reducing the risk of HCC development.

A systematic review and meta-analysis further confirmed that CHB patients who achieved HBeAg seroconversion exhibited significantly lower HCC incidence compared to those with persistent HBeAg positivity (3.37% vs. 7.4%; P = 0.02) (Zhou et al., 2018). This demonstrates that HBeAg seroconversion is not only a marker of enhanced immune control but also a pivotal factor in reducing HCC risk, thereby improving long-term outcomes for CHB patients.

### 2.2 Increasing the HBsAg seroclearance rate

HBsAg is the surface antigen of HBV and the defining serological marker of chronic HBV infection. HBsAg seroclearance refers to the loss of detectable HBsAg in serum, and it is considered the most clinically relevant surrogate of functional cure because it reflects profound suppression of viral antigen production and improved host immune control. HBeAg seroclearance and HBsAg seroclearance are linked because HBeAg loss often indicates transition from a high-replication, immune-tolerant or immune-active state toward lower viral activity, thereby creating a more favorable biological background for subsequent HBsAg decline. HBeAg seroclearance serves as a critical prerequisite for both HBeAg seroconversion and HBsAg seroclearance. In HBeAg-positive CHB patients undergoing long-term NAs therapy, a reduction in HBeAg levels at 24 weeks of treatment is associated with HBeAg seroconversion and HBsAg loss (Wong et al., 2018). A phase III study involving 266 HBeAg-positive patients receiving NAs antiviral therapy found that those who achieved successful HBeAg seroconversion by week 384 exhibited a more pronounced decline in HBeAg levels at week 24 of treatment (Wong et al., 2018). This suggests that rapid early declines in HBeAg levels may serve as an important predictor of HBeAg seroconversion and subsequent HBsAg seroclearance, offering valuable guidance for clinical treatment strategies.

Additionally, research demonstrates differential treatment responses to Peg-IFNα therapy based on HBeAg status, with HBeAg-positive patients showing reduced HBsAg clearance rates (10.3%) compared to HBeAg-negative individuals (12.5%) (Zhang et al., 2024). This disparity may stem from the immune-tolerant status of HBeAg-positive patients, high viral loads, and HBeAg-mediated suppression of immune cell function, leading to suboptimal responses to Peg-IFNα therapy. However, for CHB patients achieving HBeAg negativity and low-level HBsAg (<1500 IU/ml) on NAs, Peg-IFNα add-on therapy produced significantly superior HBsAg clearance (37.4% vs 1.9%, p<0.001) at 72 weeks (Wu et al., 2020). These findings underscore that optimizing treatment strategies, particularly through combination therapy with NAs and Peg-IFNα, can substantially enhance HBsAg clearance rates, offering new pathways and insights toward achieving clinical cure for chronic hepatitis B.

## 3 The immunological role of HBeAg

**Figure 2:**
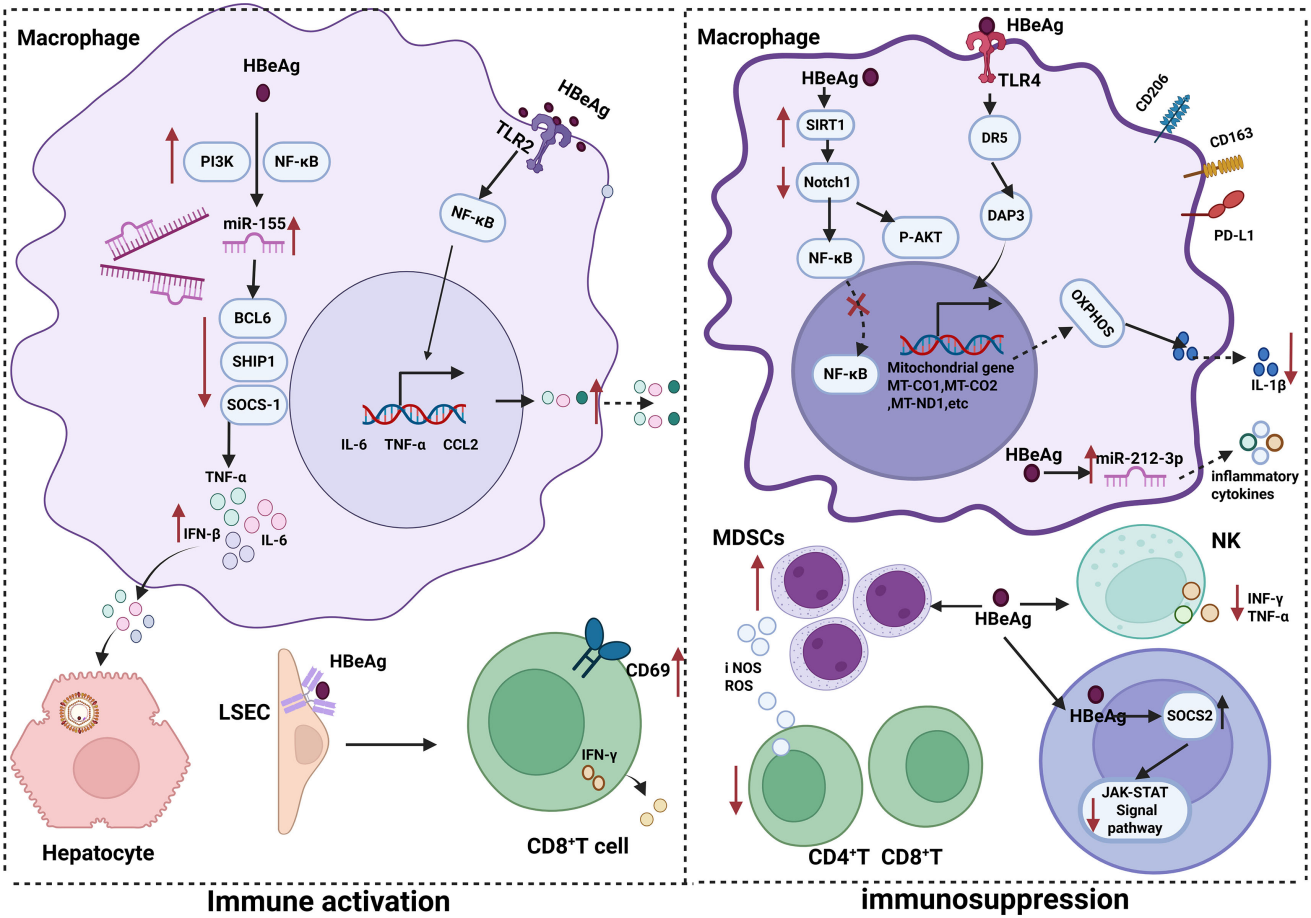
The immunological effects of HBeAg include immune activation and immunosuppression.

HBeAg, functioning as a dual-role molecule with both immunotolerogenic and immunogenic properties, exerts bidirectional immunomodulatory effects that profoundly elucidate the intricate interplay between hepatitis B virus and the host immune system (Milich, 2019). This dual regulatory characteristic transcends simple binary opposition, instead establishing dynamic equilibrium through intricate molecular mechanisms across various immune cell subsets and signaling pathways, ultimately influencing the disease progression of chronic hepatitis B (**Figure 2**).

### 3.1 HBeAg-induced immune activation

The immune activation effect of HBeAg on immune cells exhibits a classic double-edged sword phenomenon. On one hand, HBeAg activates macrophages, CD8^+^T cells, and other immune cells, triggering a cascade reaction that accelerates the progression of HBeAg-positive CHB (Xie et al., 2022). At the macrophage level, for example, HBeAg can reshape the pattern of cellular inflammatory responses by regulating the expression level of miR-155. Specifically, miR-155 enhances pro-inflammatory cytokine production by targeting inflammation-suppressing proteins such as BCL6, SHIP1, and SOCS-1, thereby exacerbating hepatocyte damage (Wang W. et al., 2018). Additionally, HBeAg activates macrophages via the TLR-2/NF-κB signaling pathway and synergistically promotes the motility, proliferation, and contraction of hepatic stellate cells (HSCs), further aggravating liver fibrosis (Xie et al., 2021b).

On the other hand, this activation also provides a potential opportunity for viral clearance. Notably, HBeAg-mediated activation of CD8^+^T cells offers novel insights for antiviral therapies. During HBV infection, liver sinusoidal endothelial cells (LSECs), as important non-professional antigen-presenting cells, have functional states that directly influence the immunogenic status of T cells and effectively relieve their inhibitory effects on T cells (Xie et al., 2021a). Animal studies have confirmed that in HBeAg gene-knockout mouse models, LSECs exhibit significantly reduced T cell activation capacity, manifested by decreased expression of the CD8^+^T cell activation marker CD69 and diminished IFN-γsecretion levels in the liver (Xie et al., 2022). These findings suggest that HBeAg plays an indispensable role in inducing specific antiviral T cell responses.

### 3.2 HBeAg-mediated immunosuppression

HBeAg remodels the hepatic immune microenvironment by multifaceted regulation of both innate and adaptive immunity, facilitating viral immune evasion and persistent infection. This immunosuppressive mechanism involves multiple critical pathways, forming an intricate regulatory network.

#### 3.2.1 Macrophage functional reprogramming

Innate immune cells, particularly macrophages with their phenotypic plasticity, are key targets of HBeAg-mediated immune regulation. HBeAg promotes the deacetylation of the Notch1 intracellular domain (NICD) by upregulating deacetylase 1 (SIRT1), thereby inhibiting NF-κB nuclear translocation and enhancing Akt phosphorylation. This drives macrophage polarization toward the immunosuppressive M2 phenotype (Li et al., 2022). Moreover, maternally derived HBeAg upregulates PD-L1 expression in hepatic macrophages and alters their polarization state upon re-stimulation with HBeAg, facilitating persistent viral infection (Tian et al., 2016). At the metabolic level, HBeAg induces the expression of death receptor 5 (DR5) and death-associated protein 3 (DAP3) via the TLR4 signaling pathway. This promotes mitochondrial gene transcription and oxidative phosphorylation (OXPHOS), impairing the antiviral functions of M1 macrophages (Li et al., 2024). In terms of cytokine regulation, HBeAg activates the MAPK pathway but concurrently induces miR-212-3p expression. Through a negative feedback mechanism, miR-212-3p suppresses pro-inflammatory cytokine production (Chen et al., 2020). Such paradoxical regulatory mechanisms further complicate the immune microenvironment.

#### 3.2.2 Regulation of signaling pathways

The interference of HBeAg with critical immune signaling pathways is a key strategy driving its immune evasion. As a direct binding receptor of HBeAg (Xie et al., 2021b), TLR-2 plays a significant role in HBV recognition (Visvanathan et al., 2007). However, the HBeAg precursor p22 protein binds to the Toll/IL-1 receptor (TIR) domain, blocking TLR signaling (Lang et al., 2011). In the JAK-STAT1 pathway, the HBeAg precursor p22 protein interacts with the nuclear transport factor (Kα1) via its C-terminal arginine-rich domain (CTD), thereby hindering the nuclear translocation of pSTAT1 and leading to impaired host innate immune responses (Mitra et al., 2019). Additionally, HBeAg suppresses IFN/JAK/STAT signal transduction at multiple levels by upregulating suppressor of cytokine signaling 2 (SOCS2). This includes reducing the stability of tyrosine kinase 2 (TYK2), downregulating IFN receptor expression, and attenuating STAT1 phosphorylation and nuclear translocation (Yu Y. et al., 2017). Further studies reveal that HBV suppresses NF-κB signaling and reduces ROS production, leading to inhibition of NLRP3 inflammasome activation and decreased IL-1β secretion, which contributes to viral persistence (Yu X. et al., 2017).

#### 3.2.3 Other innate immune cells

Among the roles in innate immune cell populations, the regulatory effects of HBeAg on natural killer (NK) cells and neutrophils significantly influence the host’s antiviral capacity. Clinical studies have shown that HBeAg-positive patients exhibit significantly lower IFN-γ secretion levels in NK cells compared to HBeAg-negative patients (de Groen et al., 2017). Concurrently, neutrophils activated by HBeAg can inhibit NK cells from producing IFN-γ and TNF-α, further exacerbating the state of immune tolerance (Feng et al., 2024). This synergistic inhibitory effect among immune cells creates a favorable environment for persistent viral infection.

#### 3.2.4 HBeAg regulation of adaptive immunity

Myeloid-derived suppressor cells (MDSCs) are immature, heterogeneous cell populations derived from myeloid progenitors, possessing potent immunosuppressive capabilities, particularly in suppressing T cell responses (Parker et al., 2015; Li et al., 2021). Studies have revealed that monocyte-derived MDSCs (mMDSCs) play a critical role in HBeAg-mediated immune tolerance. HBeAg promotes the expansion of mMDSCs and enhances their immunosuppressive functions. Research indicates that peripheral blood mMDSCs levels are significantly elevated in HBeAg-positive patients (Yang et al., 2019). These cells directly inhibit the proliferation of CD4^+^ T and CD8^+^ T cells by secreting inhibitory molecules such as arginase, iNOS and ROS (Gabrilovich and Nagaraj, 2009). Additionally, HBeAg can induce regulatory dendritic cells under immunocompromised conditions, potentially contributing to persistent infection (Lan et al., 2016). This immunosuppressive effect not only weakens the host’s specific immune response against HBV but also creates a “breeding ground” for immune evasion, facilitating viral persistence.

## 4 Factors Influencing HBeAg seroclearance

HBeAg seroclearance results from the synergistic interplay of multiple factors, including virological, host-related, and treatment-related components, which collectively form a complex regulatory network. An in-depth analysis of these factors enhances the precise understanding of the dynamic process underlying HBeAg seroclearance and provides a theoretical foundation for optimizing therapeutic strategies for CHB.

### 4.1 Virological factors

HBV DNA level, a core indicator reflecting the activity of viral replication, demonstrates a significant correlation with HBeAg seroclearance (Liu WC. et al., 2020; Hu et al., 2021). High viral load is typically associated with persistent HBeAg positivity, whereas effective antiviral therapy can markedly reduce HBV DNA levels, thereby increasing the rate of HBeAg clearance. Additionally, research indicates that quantitative hepatitis B core-related antigen (qHBcrAg), the serum quantitative assay of HBcrAg, levels measured early in Peg-IFNα treatment could help predict HBeAg seroconversion in CHB patients (Wang ML. et al., 2018). HBcrAg consists of HBeAg, hepatitis B core antigen (HBcAg), and 22-kDa precore protein (p22cr), and qHBcrAg provides a surrogate index of intrahepatic cccDNA transcriptional activity and residual viral replication. Therefore, qHBcrAg complements HBV DNA and ALT when evaluating the likelihood of HBeAg seroconversion (Testoni et al., 2019). For CHB patients undergoing NAs therapy, baseline HBcrAg levels of 6.5 log10 U/mL or lower, as well as HBcrAg levels of 5.3 log10 U/mL or lower after two years of antiviral treatment, are useful predictors of HBeAg seroconversion (Hwang et al., 2023).

HBV genotype differences also influence the progression of HBeAg seroclearance. Among the eight major globally prevalent genotypes (A-H), genotypes B and C exhibit characteristic mutations in the core promoter (CP) and pre-C regions, leading to dysregulated HBeAg expression (Liu et al., 2011). The G1896A precore mutation, commonly found in genotype C, can result in HBeAg-negative variants and increase the risk of disease progression (Lau et al., 2020). This mutation impacts viral replication and host immune responses, including cytokine production (Lau et al., 2020). HBV genotypes further affect treatment outcomes; for instance, genotype A shows a higher HBeAg seroconversion rate under interferon therapy compared to genotype C (Kumar, 2022). Understanding HBV genotype variations is critical for formulating effective management and prognostic strategies for HBV infection.

### 4.2 Host factors

There is a close relationship between the genetic polymorphisms of host genes and HBeAg seroconversion, with multiple studies in recent years revealing this association. (**Table 1**). Population-based genomic research has established that HLA-DP and HLA-DQ variants significantly influence host responses to HBV infection (Guo et al., 2011; Hu et al., 2012). These HLA genes participate in antigen presentation, and their polymorphisms may influence the efficiency of recognizing and presenting HBV antigens, thereby modulating the intensity and direction of immune responses. Further studies have shown that HLA gene variants rs2621377 (HLA-DOB) and rs3130215 (HLA-DPB2) are associated with delayed spontaneous HBeAg seroconversion in immunocompetent CHB patients (Liu WC. et al., 2020), suggesting that specific HLA locus variations may impair the host’s ability to clear HBV.

**Table 1:** Association table of host gene polymorphisms with HBeAg seroconversion.

Gene/locus	Associated phenotype	Treatment method	Study
HLA-DOB rs2621377	Significantly associated with delayed spontaneous HBeAg seroconversion in immune-active CHB patients	Natural infection	(Liu WC. et al., 2020)
HLA-DPB2 rs3130215	Significantly associated with delayed spontaneous HBeAg seroconversion in immune-active CHB patients	Natural infection	(Liu WC. et al., 2020)
IL-28B rs12980275 rs12979860 rs8099917	Independently associated with Peg-IFNα treatment response in HBeAg-positive CHB patients of Chinese Han ethnicity	Peg-IFNα therapy	(Wu H. et al., 2015)
IL-12A rs568408	Associated with HBeAg seroconversion in ETV-treated patients	ETV therapy	(Wu J. et al., 2015)
CXCR7 rs2952665	Predictor of Peg-IFNα treatment response in HBeAg-positive CHB patients	Peg-IFNα therapy	(Luo et al., 2024)
ZHX2 rs17289471	Predictor of Peg-IFNα treatment response in HBeAg-positive CHB patients	Peg-IFNα therapy	(Hou et al., 2023)
IPS1 rs6515831 rs2464	Independently associated with HBeAg seroconversion	Natural infection	(Wang et al., 2015)
STAT4 rs7574865	Associated with HBeAg seroconversion and HBsAg loss in Peg-IFNα treated patients	Peg-IFNα therapy	(Qi et al., 2022)
TANK rs3820998	Sole independent beneficial factor for achieving HBeAg seroconversion within 3 years during NAs antiviral therapy in CHB patients	NAs therapy	(Liu WC. et al., 2020)
NTCP rs7154439	Associated with HBeAg seroconversion after 48 weeks of NAs treatment	NAs therapy	(Rybicka et al., 2019)

Cytokines play a pivotal role in immune regulation, and their genetic polymorphisms are closely linked to HBV infection. For example, polymorphisms in cytokine genes such as IL-18 (Karra et al., 2015) and TNF-α (Ferreira et al., 2015) can alter cytokine expression levels and biological activity, thereby modifying antiviral immune responses. IL-12A rs568408 has been associated with HBeAg seroconversion in CHB patients treated with entecavir (ETV) (Wu J. et al., 2015), potentially by influencing Th1 cell differentiation and interferon-γ secretion to regulate antiviral immunity, though further studies are needed to elucidate the precise mechanisms. IL28B rs12979860 (CC genotype), rs12980275 (AA genotype), and rs8099917 (TT genotype) are independently associated with the response to Peg-IFNα treatment in HBeAg-positive CHB patients of the Chinese Han population (Wu H. et al., 2015). Recent research has identified CXCR7 rs2952665 and ZHX2 rs17289471 as novel predictors of Peg-IFNα treatment response (i.e., HBeAg seroconversion and HBV DNA <3.3 log IU/mL) in Chinese HBeAg-positive CHB patients (Hou et al., 2023; Luo et al., 2024).

Polymorphisms in genes related to interferon signaling pathways also significantly impact HBeAg seroconversion. The IPS1 gene variants rs6515831 TT and rs2464 CC genotypes are independently associated with HBeAg seroconversion (Wang et al., 2015), possibly by altering interferon signaling efficiency and downstream antiviral protein expression. In HBeAg-positive CHB patients treated with PegIFN-α, the STAT4 rs7574865 polymorphism correlates with HBeAg seroconversion and HBsAg loss (Qi et al., 2022), indicating its role in modulating interferon-induced immune responses. Additionally, TANK rs3820998 (CA) alone significantly enhanced the likelihood of HBeAg seroconversion within three years of NAs therapy (Liu WC. et al., 2020). The NTCP rs7154439 polymorphism is associated with HBeAg seroconversion after 48 weeks of NAs treatment (Rybicka et al., 2019). As NTCP serves as a critical receptor for HBV entry, its genetic variations may influence viral infectivity and antiviral efficacy.

In summary, host genetic polymorphism studies provide critical insights for personalized treatment of CHB patients. Screening specific genetic loci may help predict antiviral treatment responses in advance, enabling optimized therapeutic strategies to enhance HBeAg seroconversion rates. Future research should further explore interactions between genetic loci and the combined effects of gene-environment factors, offering comprehensive theoretical support for precision medicine in CHB.

### 4.3 Therapeutic factors

The selection of antiviral treatment regimens and treatment adherence are critical for achieving HBeAg seroconversion. NAs and IFN are currently the primary therapeutic agents for CHB, with distinct treatment regimens and durations significantly influencing HBeAg seroconversion rates. Peg-IFNα demonstrates unique advantages in inducing HBeAg serological conversion by modulating immune responses and directly suppressing viral replication (Woo et al., 2022). Studies indicate that Peg-IFNα monotherapy or combination therapy with NAs achieves higher HBeAg seroconversion rates, whereas long-term NAs therapy, while effective in viral suppression, yields relatively lower HBeAg seroconversion rates (Li et al., 2015; Hu et al., 2022). Tenofovir disoproxil fumarate (TDF) and tenofovir alafenamide fumarate (TAF) are potent nucleotide analogues that inhibit HBV reverse transcription/DNA polymerase activity and achieve durable HBV DNA suppression. TAF was developed to deliver tenofovir more efficiently to hepatocytes with lower systemic tenofovir exposure, thereby improving renal and bone safety in appropriate patients. Compared to the TDF group, the TAF group exhibited significantly higher HBeAg seroconversion and ALT normalization rates at 48 weeks (Wu et al., 2024), alongside improved bone mineral density and glomerular filtration rate (Buti et al., 2024), though potential dyslipidemia risks (Cao et al., 2024; Hsu et al., 2025), necessitate close lipid monitoring.

Additionally, poor patient adherence to treatment may lead to viral resistance and therapeutic failure, thereby compromising HBeAg seroconversion. Studies show that suboptimal adherence to ETV therapy (<90%) significantly increases risks of virological breakthrough, liver-related complications, and mortality (Shin et al., 2018).

## 5 Treatment and discontinuation concepts for HBeAg-negative patients

The treatment goal for HBeAg-negative patients is to achieve long-term viral suppression, reduce the risk of liver disease progression, and lower the incidence of cirrhosis and HCC. Furthermore, with effective therapeutic interventions, some patients may achieve functional cure, defined as HBsAg loss or seroconversion, undetectable HBV DNA, and resolution of liver inflammation, thereby significantly improving prognosis.

For HBeAg-negative CHB patients, NAs including ETV, TDF, TAF, or tenofovir amibufenamide (TMF) continue to serve as preferred first-line therapies, owing to their strong antiviral efficacy and acceptable safety profiles (You et al., 2023; Li Y. et al., 2025). However, NAs monotherapy rarely achieves functional cure. To enhance functional cure rates, switching to or combining Peg-IFNα with NAs therapy is an effective strategy.

A systematic review and meta-analysis demonstrated that Peg-IFNα add-on to NA therapy or switching from NA to Peg-IFNα significantly increases HBsAg loss rates compared to NA monotherapy (Liu J. et al., 2020). Peg-IFNα not only exerts direct antiviral effects but also modulates immune function, breaking immune tolerance to HBV and enabling immune-mediated clearance of HBV-infected cells, thereby promoting HBsAg loss (Zhao et al., 2024). Research has indicated that patients with baseline HBsAg levels below 1500 IU/mL and negative HBeAg status exhibit a greater probability of attaining clinical cure through sequential Peg-IFNα treatment (Ning et al., 2014; Hu et al., 2018). In such patients, the low HBsAg levels indicate relatively inactive viral replication, meaning the immune system can more easily exert its effects, thus making HBsAg clearance more probable.

Discontinuation strategies for HBeAg-negative patients remain a critical area of research. Current evidence suggests that HBsAg seroclearance rates after NAs cessation are significantly higher than with continued treatment (10.1% vs. 0%), primarily limited to patients with HBsAg <1000 IU/mL at treatment discontinuation (van Bömmel et al., 2023). Manolakopoulos et al. reported a 20% HBsAg seroclearance rate at 36 months post-NAs discontinuation, with most seroclearance occurring in patients with HBsAg <100 IU/mL at baseline (Manolakopoulos et al., 2021), underscoring the prognostic value of HBsAg levels.

Additionally, switching from NAs to Peg-IFNα for 48 weeks in HBeAg-negative CHB patients significantly reduces virological relapse rates and achieves higher HBsAg loss rates compared to NA discontinuation alone (Li F. et al., 2025). Peg-IFNα may sustain immune modulation post-therapy, enabling persistent immune control of HBV. Notably, approximately 40% of HBeAg-negative patients experience hepatitis flares after NAs discontinuation, and initiating Peg-IFNα during these flares can restore immune responses, achieving HBsAg loss in ~25% of cases (Islam et al., 2023). Thus, adjunctive Peg-IFNα therapy may still benefit eligible patients meeting NAs discontinuation criteria.

**Figure 3:**
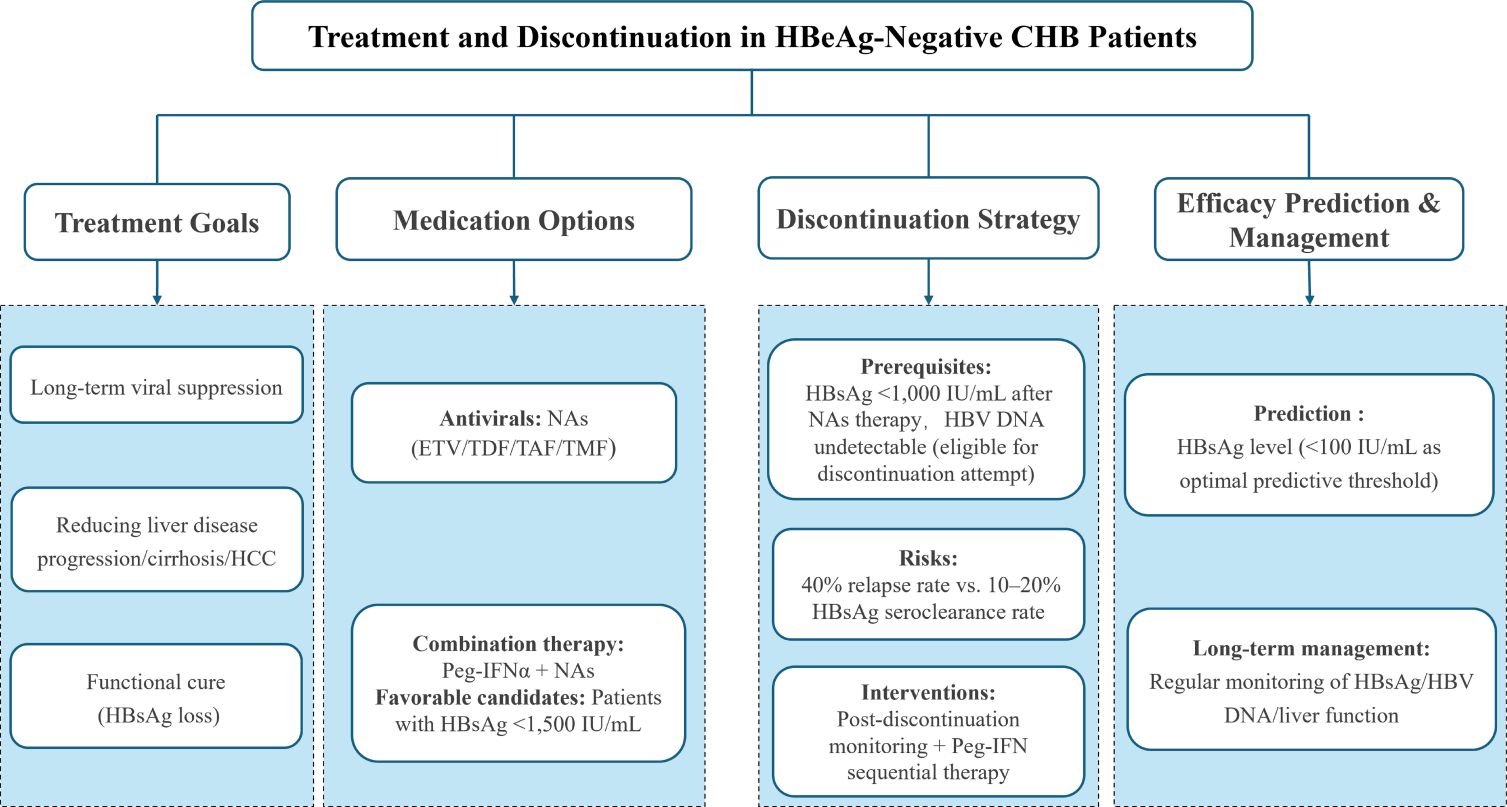
Treatment and discontinuation in HBeAg-negative CHB patients.

In summary, HBeAg-negative CHB patients meeting specific criteria (e.g., low HBsAg levels at discontinuation) exhibit higher HBsAg seroclearance rates after NAs cessation. However, close post-discontinuation monitoring is critical to optimize outcomes and mitigate relapse risks. Clinicians should individualize treatment and discontinuation strategies based on HBsAg levels, HBV DNA, liver function, and other biomarkers, with regular follow-up to detect early signs of recurrence and implement timely interventions. (**Figure 3**).

## 6 Improvement of the clinical monitoring and evaluation system

### 6.1 The application of novel biomarkers

With the continuous advancement of precision medicine, emerging novel biomarkers have provided more comprehensive tools for the clinical monitoring of CHB, particularly demonstrating significant value in predicting disease progression after HBeAg seroclearance, assessing relapse risk after treatment cessation, and evaluating long-term prognosis (Mak et al., 2019) (**Table 2**).

**Table 2:** Novel biomarkers for clinical monitoring in CHB.

Biomarker	Clinical application	Research evidence	Mechanism/characteristics
Serum HBV RNA	1. Predict HBeAg seroconversion and virological response in NAs-treated patients 2. Evaluate HBeAg seroconversion efficacy in Peg-IFNα therapy 3. Non-invasively reflect transcriptional activity of intrahepatic cccDNA 4. A pretreatment level of <4.12 log10 copies/mL is an effective predictor of HBeAg seroconversion 5. Week 12 levels predict HBeAg seroconversion and virological response at week 96	(Jia et al., 2019; Luo et al., 2019; Ji et al., 2020; Wang et al., 2021; Wang et al., 2022; Kosaka et al., 2025)	- Low levels indicate reduced cccDNA quantity or transcriptional suppression - Stronger correlation with intrahepatic cccDNA than HBcrAg, HBV DNA, etc.
HBcrAg	1. Assess viral replication and transcriptional activity during NAs therapy 2. Predict treatment outcomes (e.g., HBeAg seroconversion, HBsAg clearance)	(Lee HW. et al., 2021; Sandmann et al., 2024)	- Composed of HBV core-related proteins; reflects cccDNA activity and nucleocapsid assembly/release - Requires combined detection with traditional markers
qAnti-HBc	1. Predict HBeAg seroconversion efficacy in Peg-IFNα or NAs therapy 2. Evaluate host immune response intensity to HBV	(Hou et al., 2015; Fan et al., 2016; Shi et al., 2023; Lazarevic et al., 2024)	- Baseline >4.4 log IU/mL associated with higher seroconversion rates - Reflects hepatitis activity and liver pathological changes

HBV covalently closed circular DNA (cccDNA) has long been regarded as the gold standard for evaluating CHB cure and treatment endpoints, as its persistence represents the root cause of the difficulty in eradicating HBV infection (Yang and Kao, 2014). However, due to the invasiveness and procedural risks associated with liver biopsy, the clinical application of cccDNA quantification has faced challenges, limiting its utility as a routine monitoring indicator. Serum HBV RNA, serving as a direct reflection of cccDNA transcriptional activity, offers a novel non-invasive monitoring approach. Multiple studies have confirmed that serum HBV RNA levels are critical predictors of treatment response in NAs-treated CHB patients (Luo et al., 2019; Wang et al., 2022). Mechanistically, lower serum HBV RNA levels indicate either reduced cccDNA quantity or effectively suppressed transcriptional activity, creating favorable conditions for immune-mediated viral clearance. A study demonstrated that pre-treatment HBV RNA levels below 4.12 log10 copies/mL could serve as an effective predictor of HBeAg seroconversion (Luo et al., 2019). In high viral load patients, HBV RNA levels at week 12 of therapy could predict HBeAg seroconversion and virological response at week 96 (Ji et al., 2020). Furthermore, serum HBV RNA has also been identified as a strong predictor of HBeAg seroconversion during Peg-IFNα therapy (Jia et al., 2019; Kosaka et al., 2025). When compared to HBcrAg, HBV DNA, and HBsAg, serum HBV RNA demonstrates a more robust correlation with intrahepatic cccDNA levels both prior to and following 48 weeks of Peg-IFNα therapy (Wang et al., 2021), highlighting its potential as an optimal biomarker for reflecting cccDNA status in HBeAg-positive patients. Dynamic monitoring of serum HBV RNA enables clinicians to adjust treatment strategies promptly and implement intensive interventions for high-risk patients, thereby improving HBeAg seroconversion rates and patient outcomes.

HBcrAg, comprising HBeAg, core antigen, and 22-kDa precore protein (Lee HW. et al., 2021), demonstrates high correlation with cccDNA activity and serves as a crucial surrogate marker for evaluating viral replication and transcription. Studies have revealed that lower HBcrAg levels during NAs therapy are associated with favorable outcomes, including HBeAg seroconversion and HBsAg clearance (Sandmann et al., 2024). This correlation arises because HBcrAg not only reflects cccDNA transcriptional activity but also mirrors viral nucleocapsid assembly and release processes. Clinically, combining HBcrAg with conventional markers like HBV DNA and ALT facilitates more comprehensive disease assessment.

Quantitative anti-HBc (qAnti-HBc) levels reflect the intensity of host immune response against HBV and correlate with hepatitis activity and liver pathology (Shi et al., 2023). Clinical studies indicate that patients with baseline qAnti-HBc levels >4.4 log IU/mL receiving Peg-IFNα or NAs therapy exhibit significantly higher HBeAg seroconversion rates (Hou et al., 2015; Fan et al., 2016). A meta-analysis further confirmed that patients achieving HBeAg seroclearance had significantly higher baseline qAnti-HBc levels compared to non-responders (Lazarevic et al., 2024). In clinical practice, qAnti-HBc serves as a valuable predictive indicator for immunotherapy efficacy.

### 6.2 Dynamic monitoring and personalized treatment

Dynamic and continuous monitoring plays a critical role in optimizing treatment strategies for CHB. During therapy, regular assessment of traditional indicators such as HBeAg, HBsAg, and HBV DNA, combined with novel biomarkers, allows for more accurate evaluation of therapeutic efficacy and prediction of HBeAg seroconversion. Personalized treatment plans should be tailored based on individual patient characteristics, including age, sex, baseline viral load, degree of liver fibrosis, and genetic polymorphisms. For example, Patients with high baseline viral loads and older age may require aggressive combination therapy with intensified monitoring. Younger patients in favorable immune states could attempt more ambitious treatment strategies under close surveillance to pursue higher clinical cure rates (Terrault et al., 2018). By establishing an individualized monitoring and treatment framework, the success rate of HBeAg seroconversion can be enhanced, ultimately improving long-term patient outcomes.

## 7 Future perspectives

With the deepening of CHB research, the importance of HBeAg seroconversion in treatment has become increasingly prominent. Future treatment strategies can be optimized from the following aspects: First, deepen research on the relationship between host genetic polymorphisms and HBeAg seroconversion, leverage new technologies to identify more genetic targets, and construct precise prediction models using artificial intelligence to guide personalized therapy. Second, explore combination regimens of novel and traditional medications, determine the optimal timing, dosage, and treatment course to improve HBeAg seroconversion rates and functional cure rates. Additionally, develop modulators targeting HBeAg-mediated immune responses to break immune tolerance. Furthermore, develop novel biomarkers and establish a multi-dimensional, dynamic monitoring system to track the process of HBeAg seroconversion in real time and adjust treatment strategies promptly. Finally, improve patient follow-up systems and strengthen health education to enhance patients’ disease awareness and treatment compliance, ensuring sustained treatment efficacy.
